# Consideration Of Chronic Pain In Trials To Promote Physical Activity For Diabetes: A Systematic Review Of Randomized Controlled Trials

**DOI:** 10.1371/journal.pone.0071021

**Published:** 2013-08-07

**Authors:** John J. Riva, Jessica J. Wong, David J. Brunarski, Alice H. Y. Chan, Rebecca A. Lobo, Marina Aptekman, Mostafa Alabousi, Maha Imam, Anita Gupta, Jason W. Busse

**Affiliations:** 1 Department of Family Medicine, McMaster University, Hamilton, Ontario, Canada; 2 Department of Clinical Epidemiology and Biostatistics, McMaster University, Hamilton, Ontario, Canada; 3 Undergraduate Education, Canadian Memorial Chiropractic College, Toronto, Ontario, Canada; 4 Faculty of Health Sciences, University of Ontario Institute of Technology, Oshawa, Ontario, Canada; 5 Ontario Chiropractic Association, Toronto, Ontario, Canada; 6 Department of Family and Community Medicine, University of Toronto, Toronto, Ontario, Canada; 7 Department of Family Medicine, University of Alberta, Edmonton, Alberta, Canada; 8 Health Sciences Program, Faculty of Health Sciences, McMaster University, Hamilton, Ontario, Canada; 9 University Health Network, Toronto Rehabilitation Institute, Lyndhurst Campus, Brain and Spinal Cord Program, Toronto, Ontario, Canada; 10 Department of Anesthesia, McMaster University, Hamilton, Ontario, Canada; Postgraduate Medical Institute & Hull York Medical School, University of Hull, United Kingdom

## Abstract

**Background:**

Chronic pain has been estimated to affect 60% of patients with diabetes and is strongly associated with reduced activity tolerance. We systematically reviewed randomized controlled trials (RCTs) that explored interventions to improve physical activity among patients with diabetes to establish whether co-morbid chronic pain was captured at baseline or explored as an effect modifier and if trials reported a component designed to target chronic pain.

**Methodology/principal Findings:**

We searched CINAHL, Cochrane Central Registry of Controlled Trials, EMBASE, ERIC, MEDLINE, SPORTDiscus and PsycInfo from inception of each database to March 2012 for RCTs that enrolled patients with diabetes and randomly assigned them to an intervention designed to promote physical activity. Two reviewers independently selected trials and abstracted data. We identified 136 trials meeting our inclusion criteria, only one of which that reported capturing chronic pain measures at baseline. No trial reported on specific interventions to address chronic pain as a competing demand, or as an effect modifier.

**Conclusion/significance:**

Only 1 trial identified that aimed to promote physical activity among patients with diabetes reported that co-morbid chronic pain was captured at baseline. No trials reported exploring chronic pain as an effect modifier or targeting it as part of its intervention.

## Introduction

Physical activity is considered a cornerstone of diabetes management, along with diet and medication [1]. The benefits of physical activity are well established for patients with diabetes, especially when combined with diet [2], and include increased cardiorespiratory fitness, increased vigour, improved glycemic control, improved lipid profile and maintenance of weight loss [3]. However, chronic pain has been identified as a barrier to physical activity.

Krein et al. conducted a cross-sectional survey of 993 adult patients with diabetes (94% response rate) and found that 60% of respondents reported experiencing pain not due to cancer that was present most of the time for 6 or more months during the past year (henceforth called chronic pain). The most commonly reported pain locations involved the back (60%) and hip or knee (60%). Patients reporting chronic pain were more likely to be using insulin and to present with a higher body mass index. The presence of chronic pain was strongly associated with difficulty exercising even after adjusting for the presence of depressive symptoms, general health status, other co-morbid conditions, and priority given to diabetes care (adjusted odds ratio [OR] = 3.0; 95% confidence interval [CI] = 2.1 to 4.1) [4].

Chronic pain affecting exercise participation for type II diabetes was also described qualitatively, in patient interviews, by Casey et al., “I have difficulty… you know my knees… I have pain 24/7 in my arms and legs, in my lower back” [5]. In another study of diabetic patients, Lawton and colleagues reported numerous accounts of painful knees, joints and swollen feet. For example: “They tell you to exercise and I exercise a little, but I can’t move around a lot because I have problem with my leg [arthritis]. If I walk a little then it swells up” [6]. These forms physical pain and arthritis are frequently reported as barriers to programs of physical activity for type II diabetes [7],[8]. Lastly, two recent reviews reported both an association between neuropathic pain and impaired physical functioning [9], and that 2548 diabetic neuropathy patients across 7 identified studies have a significantly lower health-related quality of life compared to the general population (mean pooled health utility score = 0.61; 95% CI = 0.56–0.66) [10].

These findings emphasize the importance of considering chronic pain when exploring interventions targeted at increasing physical activity for patients with chronic diseases such as diabetes. We conducted a systematic review of randomized controlled trials (RCTs) to establish whether interventions aimed at increasing physical activity for patients with diabetes capture co-morbid chronic pain at baseline, if chronic pain was explored as an effect modifier, and if study interventions specifically targeted co-morbid chronic pain as a competing demand.

## Methods

Two reviewers (JJR, JWB) formulated a search strategy to identify relevant randomized controlled trials (RCTs), in English, by a systematic search of CINAHL, Cochrane Central Registry of Controlled Trials, EMBASE, ERIC, MEDLINE, SPORTDiscus and PsycInfo from inception of each database up to March 2012 (see Search Strategy S1). The search strategy combined terms for RCT, diabetes, and physical activity. The terms included free text words and subject headings specific to each database. Reviewers scanned the bibliographies of all retrieved trials and other relevant publications, including reviews and meta-analyses, for additional eligible articles.

Two teams of reviewers screened (JJR, DJB, MA, MI), independently and in duplicate, titles and abstracts of identified citations and retrieved the full text publication of articles that both reviewers judged potentially eligible. Three teams of reviewers (JJW, AHYC, RAL, MA, MA, MI) independently applied eligibility criteria to the methods section of potentially eligible trials. Eligible trials met the following criteria: (1) random allocation of patients to an intervention, including pilot studies, designed to increase physical activity or a control, and (2) inclusion of patients with type I or II diabetes. Since exercise is an effective treatment for chronic pain, any trials that incorporated primarily supervised physical activity in the domains of strength, flexibility or aerobic capacity as part of the intervention were excluded. Any disagreements were resolved by discussion to achieve consensus.

Two teams of reviewers (JJR, JJW, MA, MI) extracted data independently and in duplicate from each eligible study. Data abstracted included demographic information, methodology, intervention details (including if interventions contained components specifically targeting chronic pain), and whether co-morbid chronic pain was captured at baseline or explored as an effect modifier – either by conducting an adjusted analysis (adjusted for the presence of chronic pain, treatment group and an interaction term [chronic pain × treatment group]) or a subgroup analysis. To ensure consistency across reviewers we carried out calibration exercises before starting the review.

We assumed that consideration of chronic pain in trials to increase physical activity among patients with diabetes would be associated with: (1) starting enrolment of patients after the survey by Krein et al. was published (January 2005) [4], and (2) publication in higher impact journals, as we have recently shown that subgroup analyses are more common in high impact journals (adjusted OR = 2.64, 95% CI = 1.62 to 4.33) [11]. We reviewed the methods section of each trial, and trial registries when reported, to acquire the date that investigators began enrolling patients. We used the Institute of Scientific Information’s Journal Citation Reports to obtain the 2011 impact factor for each journal in which an eligible RCT appeared. We did not register the protocol for our review.

## Results

**Figure 1:**
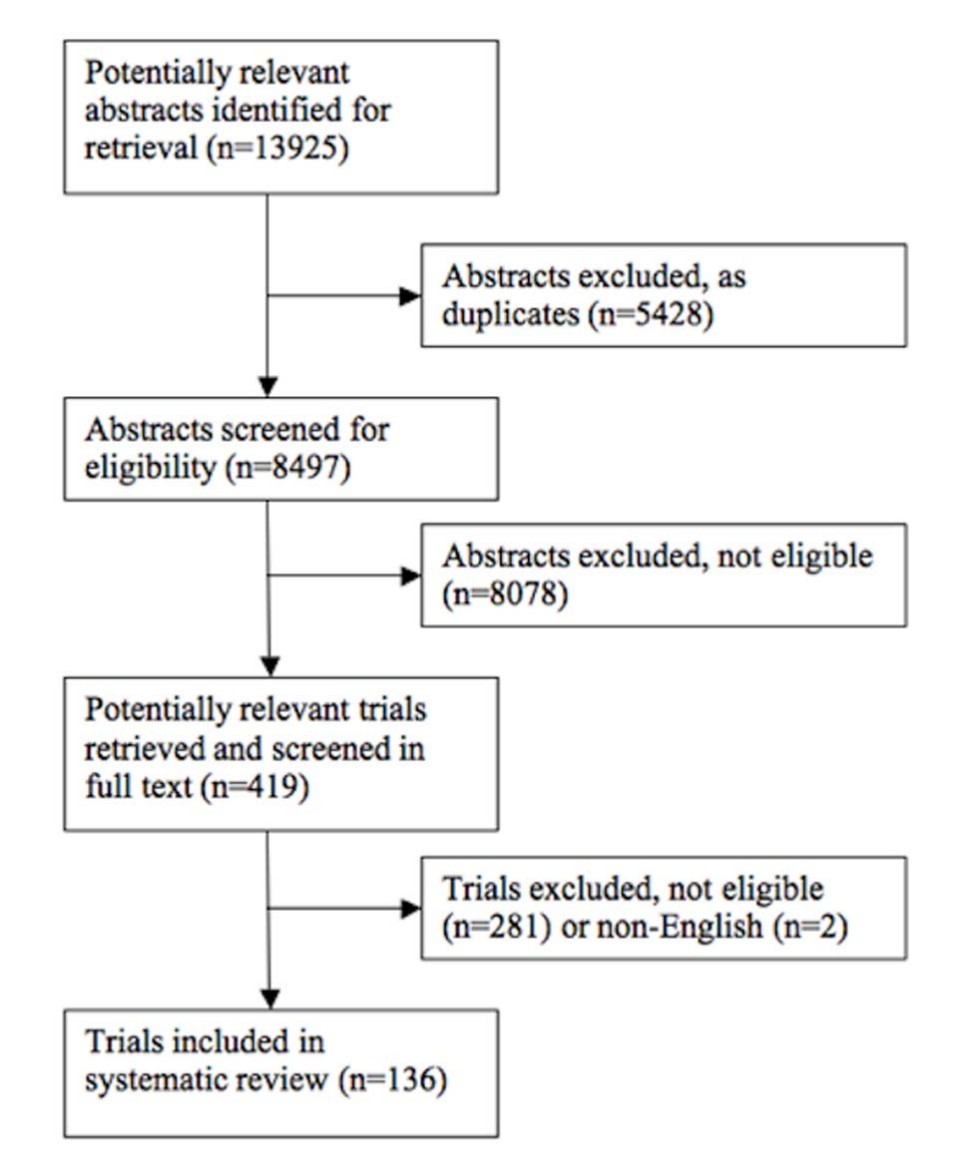
PRISMA Flow diagram showing stages of systematic review of randomized controlled trials for promoting physical activity among patients with diabetes.

We identified 13,925 potentially eligible studies, and retrieved 419 studies in full text; 136 proved eligible for our review (*see* Figure 1). The chance-adjusted between-reviewer agreement (estimated kappa) on title and abstract screening was 0.74, and 0.90 for full text eligibility. Most trials (116 of 136) enrolled patients with type II diabetes, with 9 trials enrolling patients with type I diabetes, and 11 trials enrolling mixed populations.

The majority of eligible trials (101 of 136) were published in 2006 or later; however, the date that patient enrolment began was only available for 53 of 136 trials (39%). The 2011 impact factor for listed journals ranged from 0.427 (Australian Journal of Advanced Nursing) to 11.462 (Archives of Internal Medicine).

Only 1 of the 136 eligible trials (see References Included in Review S1) recorded co-morbid chronic pain at baseline; Amoako et al. recorded that 15 of 68 participants (22%) reported chronic pain at enrolment [12]. The investigators did not conduct a statistical analysis to formally explore the effect of chronic pain on therapy, but did report that pain and stiffness were the most common reasons for patients not participating in physical activity. No trial included a component of their intervention that was explicitly directed at addressing co-morbid chronic pain. However, 28 trials did report pain-related measurements in general rather than ones specific to chronic pain, either through pain-specific measurement or as part of a general health-related quality of life instrument. (*see* Table 1).

**Table 1:** Demographic characteristics of trials that utilized pain-related measurements.

Author(s),Year	Population and Recruitment,Number (n) Enrolled	Intervention, Number(n) of Subjects	Comparisons, Number(n) of Subjects	Pain-RelatedMeasurements
Amoako *et al.* 2008	Older adult African American females from physician offices in North Carolina, United States, n = 68	Semi-structured phone interview with open-ended questions, direct exploration and use of reflective comments related to their experience with diabetes, n = 34	Usual care, which included regular primary care and specialty visits (e.g., podiatrist, eye doctor), as well as support group meetings and scheduled classes for diabetes management, n = 34	Baseline measurements for chronic pain and arthritis
Cheung et. al. 2009	Sedentary adult males and females with type II diabetes recruited from Australia, n = 37	Partially supervised group and home-based exercise sessions with resistance exercise bands, n = 20	No intervention, n = 17	SF-36: baseline and post-intervention
D’Aramo Melkus *et al.* 2010	Adult African American females with type II diabetes recruited from urban southern New England community in United States, n = 109	Culturally relevant cognitive behavioral diabetes self-management training, n = 57	Community hospital-based group diabetes education classes and group follow up sessions, n = 52	SF-36: baseline
Ell *et al.* 2010	Adult males and females with type I or II diabetes and high likelihood of clinically significant depression recruited from two public community clinics in California, United States, n = 387	Multifaceted diabetes and depression program with structured stepped-care algorithm, n = 193	Enhanced usual care consisting of standard care and depression educational pamphlets, n = 194	SF-12: baseline and post-intervention
Ell *et al.* 2011	Adult males and females with type I or II diabetes with high likelihood of clinically significant depression recruited from two public community clinics in California, United States, n = 387	Multifaceted diabetes and depression program with structured stepped-care algorithm, n = 193	Enhanced usual care consisting of standard care and depression educational pamphlets, n = 194	SF-12: baseline and post-intervention
Fritz *et al.* 2011	Overweight adult males and females with normal glucose tolerance, impaired glucose tolerance or type II diabetes recruited from Gustavsberg, Sweden, n = 212	Direction to engage in exercises, including Nordic walking with walking poles, n = 87	Control of unaltered habitual lifestyle, n = 125	One item from the Swedish Health-Related Quality of Life questionnaire measured pain at baseline and post-intervention
Gleeson-Kreig *et al.* 2006	Adult males and females with type II diabetes recruited from physician offices in New York, United States, n = 55	Kept activity records, n = 28	No activity records kept, n = 27	One item from Self-Efficacy for Exercise scale evaluating pain during exercise
Hermanns *et al.* 2012	Adult males and females with type II diabetes recruited from 18 outpatient study centers in Germany, n = 186	Diabetes education program involving intensive insulin treatment, n = 94	Established diabetes education program, n = 92	SF-12: baseline and post-intervention
Holbrook *et al.* 2009	Adult males and females with type II diabetes recruited from primary care practices in Ontario, Canada, n = 511	Web-based diabetes tracker interfaced with provider’s electronic medical record and automated telephone reminder system for the patient, n = 253	Usual care, n = 258	SF-12: baseline and post-intervention
Houweling *et al.* 2011	Adult males and females with type II diabetes referred by general practitioners to diabetes outpatient clinics in the Netherlands, n = 230	Guideline-based education and treatment provided by nurse specialized in diabetes, n = 116	Standard care (education and treatment) provided by general practitioner, n = 114	SF-36: baseline and post-intervention
Izquierdo *et al.* 2009	Male and female children (aged 5 to 14) with type I diabetes recruited from New York, United States, n = 41	Telemedicine unit in school nurse office to videoconference between the school nurse, child, and diabetes team every month, n = 23	Usual care included medical visits every 3 months and communication between school nurse and diabetes team as needed by phone, n = 18	One Dimension assessed the extent to which children experience pain during finger prick or insulin injections
Janssen *et al.* 2009	Adult males and females with screen-detected type II diabetes recruited from general practices in the Netherlands, n = 498	Intensive treatment of glucose, blood pressure and lipids with structured lifestyle education, n = 255	Routine care by general physician, n = 243	SF-36: baseline and post-intervention
Katon *et al.* 2010	Adult males and females with depression and poorly controlled diabetes (type not specified) and/or coronary heart disease recruited from 14 primary care clinics in Washington, United States, n = 214	Combined support with structured visits for self-care with pharmacotherapy to control diabetes, hyperglycemia, hypertension, and hyperlipidemia, n = 106	Enhanced usual care from primary care physician addressing depression, diabetes and/or coronary heart disease, n = 108	Overall quality of life score at baseline and post-intervention
Kuijer *et al.* 2007	Adult male and females with either type I or II diabetes recruited from hospitals in The Netherlands, n = 55	The program consisted of five tailored 2-group sessions of 6–8 patients, and was facilitated specialized nurses, n = 32	Standard care scheduled one visit (or more when needed) to the internist every year and two visits (or more when needed) to the diabetes nurse every year n = 23	Self-efficacy subscale measuring physical discomfort or pain at baseline and post-intervention
MacLean *et al.* 2009	Adult males and females with diabetes (type not specified) randomly selected from 64 primary care practices in Vermont, United States, n = 7412	Provider and patient decision support, n = 3886	Usual care, n = 3526	SF-12 and Audit of Diabetes-Dependent Quality of Life Scale measures taken post-intervention
McGowan *et al.* 2011	Adult males and females with type II diabetes recruited from diabetes education center in British Columbia, Canada, n = 321	Diabetes patient education augmented by a community self-management program, n = 169	Diabetes patient education, n = 152	One item of “Health Status” measurements accounting for pain, taken at baseline and post-intervention
O’Donnell *et al.* 2009	Adult male and females with both type II diabetes and intermittent claudication recruited from Belfast City Hospital, United Kingdom, n = 26	Using cilostazol 100mg twice a day, n = 12	Placebo, n = 14	SF-36 and Vascular Quality of Life at baseline and post-intervention. Defined claudication pain as the absolute claudication distance
Peyrot *et al.* 2010	Adult males and females with type II diabetes recruited from 29 centers in the United States, n = 119	Mealtime active inhaled insulin, n = 58	Mealtime placebo inhaled powder, n = 61	SF-36: baseline and post-intervention
Piette *et al.* 2011	Adult males and females with diabetes (type not specified) using anti-hyperglycaemic medication recruited from community, university and Veterans Affairs systems in Michigan, United States, n = 339	Telephone cognitive behavioral therapy program delivered by nurses with psychiatric and primary care training, n = 172	Enhanced usual care consisting of educational materials on self-help for depression and diabetes, n = 167	SF-12: baseline and post-intervention
Plotnikoff *et al.* 2011	Adult males and females with type II diabetes recruited from voluntary-enrollment diabetes education programs in Alberta, Canada, n = 96	Standard care (i.e. diabetes education program) supplemented with individualized counseling and community-based physical activity programs, n = 47	Standard care (i.e. diabetes education program), n = 49	Social cognitive measures taken
Rickheim *et al.* 2002	Adults with type II diabetes recruited in Minnesota, United States, n = 170	Group education in 4 sessions over 6 months, n = 87	Individual education in 4 sessions over 6 months, n = 83	SF-36: baseline and post-intervention
Rossi *et al.* 2010	Adult males and females with type I diabetes recruited from United Kingdom, Italy and Spain, n = 130	Diabetes Interactive Diary (calculator, information technology device, and telemedicine system), n = 67	Standard care and education, n = 63	SF-36: baseline and post-intervention
Rygg *et al.* 2012	Adult males and females with type II diabetes recruited from central Norway, n = 146	Diabetes self-management education delivered in group sessions, n = 73	Waiting list for one year prior to being offered diabetes self-management education group sessions, n = 73	SF-36: baseline and post-intervention
Schillinger *et al.* 2009	Adult males and females with type II diabetes recruited from California, United States, n = 339	Interactive weekly automated telephone self management support with nurse follow-up, n = 112	Monthly group medical visits with physician and health educator facilitation, n = 113 OR usual care, n = 114	SF-12: baseline and post-intervention
Sperl-Hillen *et al.* 2011	Adult males and females with type II diabetes recruited from Minnesota and New Mexico, United States, n = 623	Group education focused on diabetes self-management, n = 243	Individual education focused on diabetes self-management, n = 246 OR usual care, n = 134	SF-12: baseline and post-intervention
Tsang *et al.* 2007	Adult sedentary male and females with type II diabetes recruited through community advertising in Australia, n = 38	1-hour chair-based Tai-Chi sessions, without strength, flexibility or aerobic capacity building, once per week for 16 weeks, n = 18	Sham Tai Chi sessions, n = 20	Baseline measurements for osteoarthritis
Weinger *et al.* 2011	Adult males and females with type I or II diabetes recruited from Massachusetts, United States, n = 222	Manual-based highly structured group diabetes education program including behavioral support for implementing self-care behaviors and cognitive behavioral strategies, n = 74	Manual-based attention control group diabetes education (control condition for matching exposure to health professionals and education content), n = 75; OR unlimited individual diabetes education sessions (individual control), n = 73	Diabetes Quality of Life Questionnaire 100-point scale; measurements completed at baseline, 3, 6, and 12 months during intervention
Williamson *et al.* 2009	Overweight or obese adult males and females with type II diabetes recruited from 16 medical centers across the United States, n = 5145	Intensive lifestyle intervention including combined multiple diet and exercise approaches, n = 2570	Diabetes support and education involving educational group sessions on nutrition, physical activity and support, n = 2575	SF-36: baseline and post-intervention

doi:10.1371/journal.pone.0071021.t001

Five trials (Allen et al, 2008; Huffman et al, 2010; O’Donnell et al, 2009; Tsang et al, 2007; Van Rooljen et al, 2010) excluded patients from their trial on the basis of pain, in general, at enrolment and four trials (Bjørgaas et al, 2008; Gleeson-Kreig et al, 2006; Krousel-Wood et al, 2008; Tsang et al, 2007) recorded pain, in general, as an adverse event. Two trials otherwise mentioned pain as part of their discussion (Fritz et al, 2011; Garrett et al, 2005) (*see* Table 2). As only 1 eligible trial specifically considered chronic pain, we did not conduct our planned analyses.

**Table 2:** Pain-related exclusion criteria, adverse events and mentions.

Author(s), Year	Pain-related Exclusion Criteria and Adverse Events
Allen *et al. 2008*	Exclusion criteria: Patients with chest pain/pressure
Bjørgaas *et al.* 2008	Participants asked if they experienced chest pain or any health-related limitation for walking at each visit
Fritz *et al.* 2011	Reported increased prevalence of painful conditions of the musculoskeletal system in type II diabetics than those without type II diabetes
Garrett *et al.* 2005	Reported chronic back pain was improved by person-led self-management as it reduced patient worries and enhanced self-confidence in self-care
Huffman *et al.* 2010	Exclusion criteria: medical chart diagnosis of chronic pain preventing exercise
Krousel-Wood *et al. 2008*	Two study participants with non-cardiac chest pain events, one participant with shoulder pain
O’Donnell *et al.* 2009	Exclusion criteria: co-morbidity that limited walking capacity before the onset of claudication pain
Tsang *et al.* 2007	Exclusion criteria: severe hip or knee arthritis (causing significant pain within 30 seconds of a semi-squat position). One subject (with pre-existing spinal stenosis) in the Tai Chi group found the exercise intolerable secondary to pain and fatigue, and did not attend after session 1.
Van Rooljen *et al.* 2010	Exclusion criteria: Screened for chest pain on exertion and severe arthritis and referred to the attending specialist physician for clinical evaluation and advise about inclusion

## Discussion

Our systematic review of RCTs aimed at improving physical activity among patients with diabetes found that trials did not specifically capture co-morbid chronic pain at baseline, nor explore the impact of chronic pain as an effect modifier. Also, interventions directed at improving physical activity among patients with diabetes did not contain components specifically targeting co-morbid chronic pain as a competing demand.

Our study does have some limitations. We only considered English-language trials; however only 2 non-English trials were identified in our screening of abstracts, which suggests their exclusion, had little if any impact on our findings. As well, trials may have indeed considered aspects of chronic pain, but not reported this information. Our findings are strengthened by our comprehensive search and broad eligibility criteria, use of standardised screening and data extraction forms, and calibration exercises to enhance the consistency between reviewers.

Following the survey by Krein et al. that found 60% of patients with diabetes experience co-morbid chronic pain and suggested chronic pain should be considered as a competing demand in self-care regimes [4], Butchhart and colleagues surveyed 624 primary care patients, including 221 with diabetes (77% response rate) and found that 74% of patients with diabetes reported taking pain medications and 34% had attended a pain clinic in the last year for treatment [13]. Common locations of pain in the sample were almost identical to the findings of Krein et al.: upper back or lower back (60%), hips or knees (52%), and feet (43%). The majority of respondents (57%) managed their chronic pain with rest and reducing their level of activity and the authors noted: “It is possible that patients are not aware of the role exercise can play in pain management”.

Effective pharmacological and non-pharmacological treatments for non-specific chronic pain exist [14],[15]. Back pain was the most commonly reported chronic pain complaint in the survey by Krein et al. and the American Pain Society has recently reviewed the literature and found some evidence for tricyclic antidepressants, cognitive-behavioural therapy, exercise, spinal manipulation and interdisciplinary rehabilitation for chronic low back pain [16],[17]. Specific to diabetic neuropathic pain, a recent review identified that pregabalin and duloexitine medications are effective treatment options [18]. However, our review found that these strategies do not appear to be considered in trials exploring strategies to increase physical activity among patients with diabetes.

Chronic pain appears to be a common complaint among patients with diabetes and is associated with reduced tolerance for physical activity; however, current trials of interventions to improve physical activity in this population do not appear to consider the effect of chronic pain. When exploring interventions to improve physical activity among patients with diabetes, trialists may wish to consider baseline chronic pain and explore the impact of chronic pain as an effect modifier. Consideration could also be given to incorporating strategies within trials promoting physical activity to address complaints of co-morbid chronic pain.

## Supporting Information

Checklist S1 ****PRISMA Checklist.****
(DOC)

References Included in Review S1 (DOC)

Search Strategy S1 (DOC)
