# Supplementary material for: Consideration Of Chronic Pain In Trials To Promote Physical Activity For Diabetes: A Systematic Review Of Randomized Controlled Trials
Source: PLoS One. 2013 Aug 7;8(8):e71021. doi: 10.1371/journal.pone.0071021 (PMC3737137; doi:10.1371/journal.pone.0071021)
Supplement: References Included in Review S1 — (DOC) [file pone.0071021.s002.doc]

**Electronic Supplementary Material - References Included in Review (136)**

Al Mazroui NR, Kamal MM, Ghabash NM, Yacout TA, Kole PL, McElnay JC (2009) Influence of pharmaceutical care on health outcomes in patients with Type 2 diabetes mellitus. Br J Clin Pharmacol 67:547-57.

Allen NA, Fain JA, Braun B, Chipkin SR (2008) Continuous glucose monitoring counseling improves physical activity behaviors of individuals with type 2 diabetes: A randomized clinical trial. Diabetes Res Clin Pract 80:371-9.

Amoako E, Skelly AH, Rossen EK (2008) Outcomes of an intervention to reduce uncertainty among African American women with diabetes. West J Nurs Res 30:928-42.

Amsberg S, Anderbro T, Wredling R, Lisspers J, Lins PE et al (2009) A cognitive behavior therapy-based intervention among poorly controlled adult type 1 diabetes patients--a randomized controlled trial. Patient Educ Couns 77:72-80.

Anderson DR, Christison-Lagay J, Villagra V, Liu H, Dziura J (2010) Managing the space between visits: a randomized trial of disease management for diabetes in a community health center. J Gen Intern Med 25:1116-22.

Araiza P, Hewes H, Gashetewa C, Vella CA, Burge MR (2006) Efficacy of a pedometer-based physical activity program on parameters of diabetes control in type 2 diabetes mellitus. Metabolism 55:1382-7.

Atak N, Gurkan T, Kose K (2008) The effect of education on knowledge, self management behaviours and self efficacy of patients with type 2 diabetes. Austr J Adv Nurs 26:66-74.

Babamoto KS, Sey KA, Camilleri AJ, Karlan VJ, Catalasan J, Morisky DE (2009) Improving diabetes care and health measures among hispanics using community health workers: results from a randomized controlled trial. Health Educ Behav 36:113-26.

Barrera M Jr, Toobert DJ, Angell KL, Glasgow RE, Mackinnon DP (2006) Social support and social-ecological resources as mediators of lifestyle intervention effects for type 2 diabetes. J Health Psychol 11:483-95.

Barrera MJ, Toobert D, Strycker L, Osuna D (2012) Effects of acculturation on a culturally adapted diabetes intervention for Latinas. Health Psychol 31:51-4.

Batik O, Phelan EA, Walwick JA, Wang G, LoGerfo JP (2008) Translating a community-based motivational support program to increase physical activity among older adults with diabetes at community clinics: a pilot study of Physical Activity for a Lifetime of Success (PALS). Prev Chronic Dis 5:A18.

Belalcazar LM, Reboussin DM, Haffner SM, Hoogeveen RC, Kriska AM et al (2010) A 1-year lifestyle intervention for weight loss in individuals with type 2 diabetes reduces high C-reactive protein levels and identifies metabolic predictors of change: from the Look AHEAD (Action for Health in Diabetes) study. Diabetes Care 33:2297-303.

Best JH, Boye KS, Rubin RR, Cao D, Kim TH, Peyrot M (2009) Improved treatment satisfaction and weight-related quality of life with exenatide once weekly or twice daily. Diabet Med 26:722-8.

Bjørgaas MR, Vik JT, Stølen T, Lydersen S, Grill V (2008) Regular use of pedometer does not enhance beneficial outcomes in a physical activity intervention study in type 2 diabetes mellitus. Metabolism 57:605-11.

Boudreau F, Godin G, Poirier P (2011) Effectiveness of a computer-tailored print-based physical activity intervention among French Canadians with type 2 diabetes in a real-life setting. Health Educ Res 26:573-85.

Bradshaw BG, Richardson GE, Kumpfer K, Carlson J, Stanchfield J et al (2007) Determining the efficacy of a resiliency training approach in adults with type 2 diabetes. Diabetes Educ 33:650-9.

Cheong SH, McCargar LJ, Paty BW, Tudor-Locke C, Bell RC (2009) The First Step First Bite Program: guidance to increase physical activity and daily intake of low-glycemic index foods. J Am Diet Assoc 109:1411-6.

Cheung NW, Cinnadaio N, Russo M, Marek S (2009) A pilot randomised controlled trial of resistance exercise bands in the management of sedentary subjects with type 2 diabetes. Diabetes Res Clin Pract 83:e68-71.

Christian JG, Bessesen DH, Byers TE, Christian KK, Goldstein MG, Bock BC (2008) Clinic-based support to help overweight patients with type 2 diabetes increase physical activity and lose weight. Arch Intern Med 168:141-6.

Clark M, Hampson SE (2001) Implementing a psychological intervention to improve lifestyle self-management in patients with type 2 diabetes. Patient Educ Couns 42:247-56.

Clark M, Hampson SE, Avery L, Simpson R (2004) Effects of a tailored lifestyle self-management intervention in patients with type 2 diabetes. Br J Health Psychol 9:365-79.

Clark M, Hampson SE, Avery L, Simpson R (2009) Effects of a brief tailored intervention on the process and predictors of lifestyle behaviour change in patients with type 2 diabetes. Psychol Health Med 4:440-9.

Cleveringa FG, Minkman MH, Gorter KJ, van den Donk M, Rutten, GE (2010) Diabetes Care Protocol: effects on patient-important outcomes. A cluster randomized, non-inferiority trial in primary care. Diabet Med 27:442-50.

D'Eramo Melkus G, Chyun D, Vorderstrasse A, Newlin K, Jefferson V, Langerman S (2010) The effect of a diabetes education, coping skills training, and care intervention on physiological and psychosocial outcomes in black women with type 2 diabetes. Biol Res Nurs 12:7-19.

Davies MJ, Heller S, Skinner TC, Campbell MJ, Carey ME et al (2008) Effectiveness of the diabetes education and self management for ongoing and newly diagnosed (DESMOND) programme for people with newly diagnosed type 2 diabetes: cluster randomised controlled trial. BMJ 336:491-5.

De Greef K, Deforche B, Tudor-Locke C, De Bourdeaudhuij I (2011) Increasing physical activity in Belgian type 2 diabetes patients: a three-arm randomized controlled trial. Int J Behav Med 18:188-98.

De Greef KP, Deforche BI, Ruige JB, Bouckaert JJ, Tudor-Locke CE et al (2011) The effects of a pedometer-based behavioral modification program with telephone support on physical activity and sedentary behavior in type 2 diabetes patients. Patient Educ Couns 84:275-9.

Deakin TA, Cade JE, Williams R, Greenwood DC (2006) Structured patient education: the diabetes X-PERT Programme makes a difference. Diabet Med 23:944-54.

DeGreef K, Deforche B, Tudor-Locke C, De Bourdeaudhuij I (2010) A cognitive-behavioural pedometer-based group intervention on physical activity and sedentary behaviour in individuals with type 2 diabetes. Health Edu Res 25:724-36.

Di Loreto C, Fanelli C, Lucidi P, Murdolo G, De Cicco A et al (2003) Validation of a counseling strategy to promote the adoption and the maintenance of physical activity by type 2 diabetic subjects. Diabetes Care 26:404-8.

Doucette WR, Witry MJ, Farris KB, McDonough RP (2009) Community pharmacist-provided extended diabetes care. Ann Pharmacother 43:882-9.

Dutton GR, Provost BC, Tan F, Smith D (2008) A tailored print-based physical activity intervention for patients with type 2 diabetes. Prev Med 47:409-11.

Dyson PA, Beatty S, Matthews DR (2010) An assessment of lifestyle video education for people newly diagnosed with type 2 diabetes. J Hum Nutr Diet 23:353-9.

Eakin E, Reeves M, Lawler S, Graves N, Oldenburg B et al (2009) Telephone counseling for physical activity and diet in primary care patients. Am J Prev Med 36:142-9.

Ell K, Katon W, Xie B, Lee P, Kapetanovic S et al (2010) Collaborative care management of major depression among low-income, predominantly Hispanic subjects with diabetes: A randomized controlled trial. Diabetes Care 33:706-13.

Ell K, Katon W, Xie B, Lee P, Kapetanovic S et al (2011) One-year postcollaborative depression care trial outcomes among predominantly Hispanic diabetes safety net patients. Gen Hosp Psychiatry 33:436-42.

Engel L, Lindner H (2006) Impact of using a pedometer on time spent walking in older adults with type 2 diabetes. Diabetes Educ 32:98-107.

Estabrooks PA, Nelson CC, Xu S, King D, Bayliss EA et al (2005) The frequency and behavioral outcomes of goal choices in the self-management of diabetes. Diabetes Educ 31:391-400.

Faridi Z, Liberti L, Shuval K, Northrup V, Ali A, Katz DL (2008) Evaluating the impact of mobile telephone technology on type 2 diabetic patients' self-management: the NICHE pilot study. J Eval Clin Pract 14:465-9.

Fritz T, Caidahl K, Osler M, Ostenson CG, Zierath JR, Wandell P (2011) Effects of Nordic walking on health-related quality of life in overweight individuals with type 2 diabetes mellitus, impaired or normal glucose tolerance. Diabet Med 28:1362-72.

Frosch DL, Uy V, Ochoa S, Mangione CM (2011) Evaluation of a behavior support intervention for patients with poorly controlled diabetes. Arch Intern Med 171:2011-7.

Gaede P, Beck M, Vedel P, Pedersen O (2001) Limited impact of lifestyle education in patients with Type 2 diabetes mellitus and microalbuminuria: results from a randomized intervention study. Diabet Med 18:104-8.

Garrett N, Hageman CM, Sibley SD, Davern M, Berger M et al (2005) The effectiveness of an interactive small group diabetes intervention in improving knowledge, feeling of control, and behavior. Health Promot Pract 6:320-8.

Glasgow RE, Boles SM, McKay HG, Feil EG, Barrera M Jr (2003) The D-Net diabetes self-management program: long-term implementation, outcomes, and generalization results. Prev Med 36:410-9.

Glasgow RE, Christiansen SM, Kurz D, King DK, Woolley T et al (2011) Engagement in a diabetes self-management website: usage patterns and generalizability of program use. J Med Internet Res 13:e9.

Glasgow RE, Kurz D, King D, Dickman JM, Faber AJ et al (2010) Outcomes of minimal and moderate support versions of an internet-based diabetes self-management support program. J Gen Intern Med 25:1315-22.

Glasgow RE, Strycker LA, King DK, Toobert DJ, Rahm AK et al (2006) Robustness of a computer-assisted diabetes self-management intervention across patient characteristics, healthcare settings, and intervention staff. Am J Manag Care 12:137-45.

Gleeson-Kreig JM (2006) Self-monitoring of physical activity: effects on self-efficacy and behavior in people with type 2 diabetes. Diabetes Educ 32:69-77.

Hanefeld M, Fischer S, Schmechel H, Rothe G, Schulze J et al (1991) Multi-intervention trial in newly diagnosed NIDDM. Diabetes Care 14:308-17.

Hasler TD, Fisher BM, MacIntyre PD, Mutrie N (2000) Exercise consultation and physical activity in patients with type 1 diabetes. Practical Diabetes Int 17:44-8.

Heinrich E, Candel MJ, Schaper NC, de Vries NK (2010) Effect evaluation of a Motivational Interviewing based counselling strategy in diabetes care. Diabetes Res Clin Pract 90:270-8.

Hermanns N, Kulzer B, Maier B, Mahr M, Haak T (2012) The effect of an education programme (MEDIAS 2 ICT) involving intensive insulin treatment for people with type 2 diabetes. Patient Educ Couns 86:226-32.

Holbrook A, Thabane L, Keshavjee K, Dolovich L, Bernstein B et al (2009) Individualized electronic decision support and reminders to improve diabetes care in the community: COMPETE II randomized trial. CMAJ 181:37-44.

Horan PP, Yarborough MC, Besigel G, Carlson DR (1990) Computer-assisted self-control of diabetes by adolescents. Diabetes Educ 16:205-11.

Houweling ST, Kleefstra N, van Hateren KJ, Groenier KH, Meyboom-de Jong B, Bilo HJ (2011) Can diabetes management be safely transferred to practice nurses in a primary care setting? A randomised controlled trial. J Clin Nurs 20:1264-72.

Huang JP, Chen HH, Yeh ML (2009) A comparison of diabetes learning with and without interactive multimedia to improve knowledge, control, and self-care among people with diabetes in Taiwan. Public Health Nurs 26:317-28.

Huffman KM, Sloane R, Peterson MJ, Bosworth HB, Ekelund C et al (2010) The impact of self-reported arthritis and diabetes on response to a home-based physical activity counselling intervention. Scand J Rheumatol 39:233-9.

Huisman S, DeGucht V, Maes S, Schroevers M, Chatrou M, Haak H (2009) Self-regulation and weight reduction in patients with type 2 diabetes: a pilot intervention study. Patient Educ Couns 75:84-90.

Izquierdo R, Morin PC, Bratt K, Moreau Z, Meyer S et al (2009) School-centered telemedicine for children with type 1 diabetes mellitus. J Pediatr 155:374-9.

Jackson R, Asimakopoulou K, Scammell A (2007) Assessment of the transtheoretical model as used by dietitians in promoting physical activity in people with type 2 diabetes. J Hum Nutr Diet 20:27-36.

Jakicic JM, Jaramillo SA, Balasubramanyam A, Bancroft B, Curtis JM et al (2009) Effect of a lifestyle intervention on change in cardiorespiratory fitness in adults with type 2 diabetes: results from the Look AHEAD Study. Int J Obes (Lond) 33:305-16.

Janssen PG, Gorter KJ, Stolk RP, Rutten GE (2009) Randomised controlled trial of intensive multifactorial treatment for cardiovascular risk in patients with screen-detected type 2 diabetes: 1-year data from the ADDITION Netherlands study. Br J Gen Pract 59:43-8.

Katon WJ, Lin EH, Von Korff M, Ciechanowski P, Ludman EJ et al (2010) Collaborative care for patients with depression and chronic illnesses. N Engl J Med 363:2611-20.

Kattelmann KK, Conti K, Ren C (2009) The medicine wheel nutrition intervention: a diabetes education study with the Cheyenne River Sioux Tribe. J Am Diet Assoc 109:1532-9.

Keyserling TC, Samuel-Hodge CD, Ammerman AS, Ainsworth BE, Henríquez-Roldán CF et al (2002) A randomized trial of an intervention to improve self-care behaviors of African-American women with type 2 diabetes: impact on physical activity. Diabetes Care 25:1576-83.

Kim C-J, Kang D-H (2006) Utility of a web-based intervention for individuals with type 2 diabetes: the impact on physical activity levels and glycemic control. Comput Inform Nurs 24:337-45.

Kim G-J, Hwang A-R (2004) The impact of a stage-matched intervention to promote exercise behavior in participants with type 2 diabetes. Int J Nurs Stud 41:833-41.

Kim H-S, Oh J-A (2003) Adherence to diabetes control recommendations: impact of nurse telephone calls. Journal of Advanced Nursing 44:256–61.

King DK, Estabrooks PA, Strycker LA, Toobert DJ, Bull SS, Glasgow RE (2006) Outcomes of a multifaceted physical activity regimen as part of a diabetes self-management intervention. Ann Behav Med 31:128-37.

Kirk A, Barnett J, Leese G, Mutrie N (2009) A randomized trial investigating the 12-month changes in physical activity and health outcomes following a physical activity consultation delivered by a person or in written form in Type 2 diabetes: Time2Act. Diabet Med 26:293-301.

Kirk A, Mutrie N, MacIntyre P, Fisher M (2003) Increasing physical activity in people with type 2 diabetes. Diabetes Care 26:1186-92.

Kirk A, Mutrie N, MacIntyre P, Fisher M (2004) Effects of a 12-month physical activity counselling intervention on glycaemic control and on the status of cardiovascular risk factors in people with Type 2 diabetes. Diabetologia 47:821-32.

Kirk AF, Higgins LA, Hughes AR, Fisher BM, Mutrie N et al (2001) A randomized, controlled trial to study the effect of exercise consultation on the promotion of physical activity in people with Type 2 diabetes: a pilot study. Diabet Med 18:877-82.

Kirk AF, Mutrie N, Macintyre PD, Fisher MB (2004) Promoting and maintaining physical activity in people with type 2 diabetes. Am J Prev Med 27:289-96.

Kirkman MS, Weinberger M, Landsman PB, Samsa GP, Shortliffe EA et al (1994) A telephone-delivered intervention for patients with NIDDM. Effect on coronary risk factors. Diabetes Care 17:840-6.

Krousel-Wood MA, Berger L, Jiang X, Blonde L, Myers L, Webber L (2008) Does home-based exercise improve body mass index in patients with type 2 diabetes? results of a feasibility trial. Diabetes Res Clin Pract 79:230-6.

Kuijer RG, De Ridder DTD, Colland VT, Schreurs KMG, Sprangers MAG (2007) Effects of a short self-management intervention for patients with asthma and diabetes: Evaluating health-related quality of life using then-test methodology. Psychology & Health 22:387-411.

Kulzer B, Hermanns N, Reinecker H, Haak T (2007) Effects of self-management training in Type 2 diabetes: a randomized, prospective trial. Diabet Med 24:415-423.

Lee A, Siu CF, Leung KT, Lau LC, Chan CC, Kwok KW (2011) General practice and social service partnership for better clinical outcomes, patient self efficacy and lifestyle behaviours of diabetic care: randomised control trial of a chronic care model. Postgrad Med J 87:688-93.

Lee LJ, Fahrbach JL, Nelson LM, McLeod LD, Martin SA et al (2010) Effects of insulin initiation on patient-reported outcomes in patients with type 2 diabetes: results from the durable trial. Diabetes Res Clin Pract 89:157-66.

Liebreich T, Plotnikoff RC, Courneya KS, Boulé N (2009) Diabetes NetPLAY: A physical activity website and linked email counselling randomized intervention for individuals with type 2 diabetes. Int J Behav Nutr Phys Act 6:18.

Lin EH, Katon W, Rutter C, Simon GE, Ludman EJ et al (2006) Effects of enhanced depression treatment on diabetes self-care. Ann Fam Med 4:46-53.

Logtenberg SJ, Kleefstra N, Houweling ST, Groenier KH, Gans RO, Bilo HJ (2010) Health-related quality of life, treatment satisfaction, and costs associated with intraperitoneal versus subcutaneous insulin administration in type 1 diabetes: a randomized controlled trial. Diabetes Care 33:1169-72.

Look AHEAD Research Group, Wing RR (2010) Long-term effects of a lifestyle intervention on weight and cardiovascular risk factors in individuals with type 2 diabetes mellitus: four-year results of the Look AHEAD trial. Arch Intern Med 170:1566-75.

Lorig K, Ritter PL, Laurent DD, Plant K, Green M et al (2010) Online diabetes self-management program: a randomized study. Diabetes Care 33:1275-81.

Lorig K, Ritter PL, Villa FJ, Armas J (2009) Community-based peer-led diabetes self-management: a randomized trial. Diabetes Educ 35:641-51.

MacLean CD, Gagnon M, Callas P, Littenberg B (2009) The Vermont diabetes information system: A cluster randomized trial of a population based decision support system. J Gen Intern Med 24:1303-10.

Malpass A, Andrews R, Turner KM (2009) Patients with Type 2 Diabetes experiences of making multiple lifestyle changes: a qualitative study. Patient Educ Couns 74:258-63.

McGowan P (2011) The efficacy of diabetes patient education and self-management education in type 2 diabetes. Canadian Journal of Diabetes 35:46-53.

McKay HG, King D, Eakin EG, Seeley JR, Glasgow RE (2001) The diabetes network internet-based physical activity intervention: a randomized pilot study. Diabetes Care 24:1328-34.

Mehuys E, Van Bortel L, De Bolle L, Van Tongelen I, Annemans L et al (2011) Effectiveness of a community pharmacist intervention in diabetes care: a randomized controlled trial. J Clin Pharm Ther 36:602-13.

Ménard J, Payette H, Baillargeon JP, Maheux P, Lepage S et al (2005) Efficacy of intensive multitherapy for patients with type 2 diabetes mellitus: a randomized controlled trial. CMAJ 173:1457-66.

Ménard J, Payette H, Dubuc N, Baillargeon JP, Maheux P, Ardilouze JL (2007) Quality of life in type 2 diabetes patients under intensive multitherapy. Diabetes Metab 33:54-60.

Nesari M, Zakerimoghadam M, Rajab A, Bassampour S, Faghihzadeh S (2010) Effect of telephone follow-up on adherence to a diabetes therapeutic regimen. Jpn J Nurs Sci 7:121-8.

Newton KH, Wiltshire EJ, Elley CR (2009) Pedometers and text messaging to increase physical activity: randomized controlled trial of adolescents with type 1 diabetes. Diabetes Care 32:813-5.

Ng CL, Tai ES, Goh SY, Wee HL (2011) Health status of older adults with Type 2 diabetes mellitus after aerobic or resistance training: a randomised trial. Health Qual Life Outcomes 9: 59.

Nunn E, King B, Smart C, Anderson D (2006) A randomized controlled trial of telephone calls to young patients with poorly controlled type 1 diabetes. Pediatr Diabetes 7:254-9.

O'Donnell ME, Badger SA, Sharif MA, Makar RR, Young IS et al (2009) The vascular and biochemical effects of cilostazol in diabetic patients with peripheral arterial disease. Vasc Endovascular Surg 43:132-43.

Osborn CY, Amico KR, Cruz N, O'Connell AA, Perez-Escamilla R et al (2010) A brief culturally tailored intervention for Puerto Ricans with type 2 diabetes. Health Educ Behav 37:849-62.

Paschali AA, Goodrick GK, Kalantzi-Azizi A, Papadatou D, Balasubramanyam A (2005) Accelerometer feedback to promote physical activity in adults with type 2 diabetes: a pilot study. Percept Mot Skills 100:61-8.

Peyrot M, Rubin RR (2010) Effect of technosphere inhaled insulin on quality of life and treatment satisfaction. Diabetes Technol Ther 12:49-55.

Piette JD, Richardson C, Himle J, Duffy S, Torres T et al (2011) A randomized trial of telephonic counseling plus walking for depressed diabetes patients. Med Care 49:641-8.

Plotnikoff RC, Pickering MA, Glenn N, Doze SL, Reinbold-Matthews ML et al (2011) The effects of a supplemental, theory-based physical activity counseling intervention for adults with type 2 diabetes. J Phys Act Health 8:944-54.

Richardson CR, Mehari KS, McIntyre LG, Janney AW, Fortlage LA et al (2007) A randomized trial comparing structured and lifestyle goals in an internet-mediated walking program for people with type 2 diabetes. Int J Behav Nutr Phys Act 4:59.

Rickheim PL, Weaver TW, Flader JL, Kendall DM (2002) Assessment of group versus individual diabetes education: a randomized study. Diabetes Care 25:269-74.

Rossi MC, Nicolucci A, Di Bartolo P, Bruttomesso D, Girelli A et al (2010) Diabetes Interactive Diary: a new telemedicine system enabling flexible diet and insulin therapy while improving quality of life: an open-label, international, multicenter, randomized study. Diabetes Care 33:109-15.

Rygg LØ, Rise MB, Gronning K, Steinsbekk A (2012) Efficacy of ongoing group based diabetes self-management education for patients with type 2 diabetes mellitus: a randomised controlled trial. Patient Educ Couns 86:98-105.

Sacco WP, Malone JI, Morrison AD, Friedman A, Wells K (2009) Effect of a brief, regular telephone intervention by paraprofessionals for type 2 diabetes. J Behav Med 32:349-59.

Sadur CN, Moline N, Costa M, Michalik D, Mendlowitiz D et al (1999) Diabetes management in a health maintenance organization. Efficacy of care management using cluster visits. Diabetes Care 22:2011-7.

Samuel-Hodge CD, Keyserling TC, Park S, Johnston LF, Gizlice Z, Bangdiwala SI (2009) A randomized trial of a church-based diabetes self-management program for African Americans with type 2 diabetes. Diabetes Educ 35:439-54.

Schillinger D, Wang F, Handley M, Hammer H (2009) Effects of self-management support on structure, process, and outcomes among vulnerable patients with diabetes. Diabetes Care 32:559-66.

Smith DE, Heckemeyer CM, Kratt PP, Mason DA (1997) Motivational interviewing to improve adherence to a behavioral weight-control program for older obese women with NIDDM. A pilot study. Diabetes Care 20:52-4.

Sperl-Hillen J, Beaton S, Fernandes O, Von WA, Vazquez-Benitez G et al (2011) Comparative effectiveness of patient education methods for type 2 diabetes: a randomized controlled trial. Arch Intern Med 171:2001-10.

Steed L, Lankester J, Barnard M, Earle K, Hurel S, Newman S (2005) Evaluation of the UCL diabetes self-management programme (UCL-DSMP): a randomized controlled trial. J Health Psychol 10:261-76.

Tan MY, Magarey JM, Chee SS, Lee LF, Tan MH (2011) A brief structured education programme enhances self-care practices and improves glycaemic control in Malaysians with poorly controlled diabetes. Health Educ Res 26:896-907.

Thoolen BJ, de Ridder D, Bensing J, Gorter K, Rutten G (2009) Beyond good intentions: The role of proactive coping in achieving sustained behavioural change in the context of diabetes management. Psychol Health 24:237-54.

Toobert DJ, Glasgow RE, Strycker LA, Barrera M Jr, Ritzwoller DP, Weidner G (2007) Long-term effects of the Mediterranean lifestyle program: a randomized clinical trial for postmenopausal women with type 2 diabetes. Int J Behav Nutr Phys Act 4:1.

Toobert DJ, Strycker LA, Barrera M Jr, Osuna D, King DK, Glasgow RE (2011) Outcomes from a multiple risk factor diabetes self-management trial for Latinas: ¡Viva Bien! Ann Behav Med 41:310-23.

Tsang T, Orr R, Lam P, Comino EJ, Singh MF (2007) Health benefits of Tai Chi for older patients with type 2 diabetes: the "Move It For Diabetes study"--a randomized controlled trial. Clin Interv Aging 2:429-39.

Tu KS, McDaniel G, Gay JT (1993) Diabetes self-care knowledge, behaviors, and metabolic control of older adults--the effect of a posteducational follow-up program. Diabetes Educ 19:25-30.

Tudor-Locke C, Bell RC, Myers AM, Harris SB, Ecclestone NA et al (2004) Controlled outcome evaluation of the First Step Program: a daily physical activity intervention for individuals with type II diabetes. Int J Obes Relat Metab Discord 28:113-9.

Uusitupa MI (1996) Early lifestyle intervention in patients with non-insulin-dependent diabetes mellitus and impaired glucose tolerance. Ann Med 28:445-9.

Van Rooljen A, Viviers CM, Becker PJ (2010) A daily physical activity and diet intervention for individuals with type 2 diabetes mellitus: a randomized controlled trial. SA J Physiotherapy 66:9-16.

Vanninen E, Uusitupa M, Siitonen O, Laitinen J, Länsimies E (1992) Habitual physical activity, aerobic capacity and metabolic control in patients with newly-diagnosed type 2 (non-insulin-dependent) diabetes mellitus: effect of 1-year diet and exercise intervention. Diabetologia 35:340-6.

Wadden TA, West DS, Neiberg RH, Wing RR, Ryan DH et al (2009) One-year weight losses in the Look AHEAD study: factors associated with success. Obesity (Silver Spring) 17:713-22.

Wangberg SC (2008) An Internet-based diabetes self-care intervention tailored to self-efficacy. Health Educ Res 23:170-9.

Watanabe M, Okayama A, Shimamoto K, Ueshima H (2007) Short-term effectiveness of an individual counseling program for impaired fasting glucose and mild type 2 diabetes in Japan: a multi-center randomized control trial. Asia Pac J Clin Nutr 16:489-97.

Weinger K, Beverly EA, Lee Y, Sitnokov L, Ganda OP, Caballero AE (2011) The effect of a structured behavioral intervention on poorly controlled diabetes: a randomized controlled trial. Arch Intern Med 171:1990-9.

Weinstock RS, Brooks G, Palmas W, Morin PC, Teresi JA et al (2011) Lessened decline in physical activity and impairment of older adults with diabetes with telemedicine and pedometer use: results from the IDEATel study. Age Ageing 40:98-105.

Whittemore R, Melkus GD, Sullivan A, Grey M (2004) A nurse-coaching intervention for women with type 2 diabetes. Diabetes Educ 30:795-804.

Williamson DA, Rejeski J, Lang W, Van Dorsten B, Fabricatore AN, Toledo K; Look AHEAD Research Group (2009) Impact of a weight management program on health-related quality of life in overweight adults with type 2 diabetes. Arch Intern Med 169:163-71.

Wing RR, Epstein LH, Nowalk MP, Koeske R, Hagg S (1985) Behavior change, weight loss, and physiological improvements in type II diabetic patients. J Consult Clin Psychol 53:111-22.

Wolever RQ, Dreusicke M, Fikkan J, Hawkins TV, Yeung S et al (2010) Integrative health coaching for patients with type 2 diabetes: a randomized clinical trial. Diabetes Educ 36:629-39.

Wong FK, Mok MP, Chan T, Tsang MW (2005) Nurse follow-up of patients with diabetes: randomized controlled trial. J Adv Nurs 50:391-402.

Woo J, Sea MM, Tong P, Ko GT, Lee Z et al (2007) Effectiveness of a lifestyle modification programme in weight maintenance in obese subjects after cessation of treatment with Orlistat. J Eval Clin Pract 13:853-9.

Yoo HJ, An HG, Park SY, Ryu OH, Kim HY et al (2008) Use of a real time continuous glucose monitoring system as a motivational device for poorly controlled type 2 diabetes. Diabetes Res Clin Pract 82:73-9.
