# Supplementary material for: Consideration Of Chronic Pain In Trials To Promote Physical Activity For Diabetes: A Systematic Review Of Randomized Controlled Trials
Source: PLoS One. 2013 Aug 7;8(8):e71021. doi: 10.1371/journal.pone.0071021 (PMC3737137; doi:10.1371/journal.pone.0071021)
Supplement: Search Strategy S1 — (DOC) [file pone.0071021.s003.doc]

**Supplementary Material - Search Strategy to March 2012**

CINAHL (1476)

((MH "Diabetes Mellitus, Insulin-Dependent") OR (MH "Diabetes Mellitus, Non-Insulin-Dependent") OR (MH "Diabetes Mellitus")) AND ("exercise" OR "physical activity" OR “self care" OR “quality of life” OR “intervention” OR “interventions” OR “lifestyle” OR “motivational interviewing” OR “self-management”) AND (“random*” OR “controlled”)

SPORTSDiscuss (300)

MEDLINE 1996 - present (4544)

EMBASE 1980 - present (4007)

PsychINFO 1967 - present (1556)

(Diabetes OR Diabetes Mellitus) AND (“physical activity” OR “self management” OR “exercise” OR “self efficacy” OR “lifestyle changes” OR “obesity” OR “quality of life” OR “intervention” OR “interventions” OR “lifestyle” OR “motivational interviewing” OR “self-management” OR “quality of life” OR “exercise counseling”)

ERIC (198)

diabet* AND (physical activit* OR physical fitness OR self efficacy OR exercise)

COCHRANE CONTROLLED TRIALS (1844)

(diabetes OR diabetic) AND (“physical activity” OR “physical fitness” OR “exercise” OR “self care” OR “self management” OR “intervention” OR “self efficacy” OR “quality of life”)

TOTAL citations across all databases (13925)
